# An APC/C-Cdh1 Biosensor Reveals the Dynamics of Cdh1 Inactivation at the G1/S Transition

**DOI:** 10.1371/journal.pone.0159166

**Published:** 2016-07-13

**Authors:** Andrej Ondracka, Jonathan A. Robbins, Frederick R. Cross

**Affiliations:** Laboratory of Cell Cycle Genetics, The Rockefeller University, New York, NY 10065, United States of America; University College London, UNITED KINGDOM

## Abstract

B-type cyclin-dependent kinase activity must be turned off for mitotic exit and G1 stabilization. B-type cyclin degradation is mediated by the anaphase-promoting complex/cyclosome (APC/C); during and after mitotic exit, APC/C is dependent on Cdh1. Cdh1 is in turn phosphorylated and inactivated by cyclin-CDK at the Start transition of the new cell cycle. We developed a biosensor to assess the cell cycle dynamics of APC/C-Cdh1. Nuclear exit of the G1 transcriptional repressor Whi5 is a known marker of Start; APC/C-Cdh1 is inactivated 12 min after Whi5 nuclear exit with little measurable cell-to-cell timing variability. Multiple phosphorylation sites on Cdh1 act in a redundant manner to repress its activity. Reducing the number of phosphorylation sites on Cdh1 can to some extent be tolerated for cell viability, but it increases variability in timing of APC/C-Cdh1 inactivation. Mutants with minimal subsets of phosphorylation sites required for viability exhibit striking stochasticity in multiple responses including budding, nuclear division, and APC/C-Cdh1 activity itself. Multiple cyclin-CDK complexes, as well as the stoichiometric inhibitor Acm1, contribute to APC/C-Cdh1 inactivation; this redundant control is likely to promote rapid and reliable APC/C-Cdh1 inactivation immediately following the Start transition.

## Introduction

The main driver of the eukaryotic cell cycle is the periodic rise and fall of the cyclin-dependent kinase (CDK) activity [1]. The anaphase-promoting complex/cyclosome (APC/C) [2,3] drives ubiquitylation and degradation of mitotic cyclins as well as multiple other cell cycle regulators [4].

Ubiquitinylation by the mitotic APC/C requires the binding of one of two activators, Cdc20 and Cdh1. APC/C-Cdc20 is active at the metaphase-anaphase transition. Its targets in the budding yeast *Saccharomyces cerevisiae* include the S-phase cyclin Clb5, the mitotic cyclin Clb2, and the securin protein Pds1 [5–8]. In contrast, APC/C-Cdh1 is activated later, completing mitotic exit and stabilizing the G1 phase by preventing premature accumulation of mitotic cyclins [9,10]. Its targets also include the polo kinase Cdc5 [8,11,12], various components of the mitotic spindle [13,14], Cdc20 [15], and a mitotic transcription factor Ndd1 [16]. Since its targets are important mitotic activators, proper progression through the cell cycle therefore requires inactivation of APC/C-Cdh1 throughout mitosis.

The cell cycle Start marks the irreversible commitment to a new cell cycle [17]. The commitment to a new cell cycle is achieved by a transcriptional positive feedback loop of the G1 cyclins, in large part involving phosphorylation of the inhibitor Whi5 [18]. This feedback loop ensures irreversibility [19] and coherent and orderly expression of the G1/S genes that promote budding and S-phase [18,20], driven by MBF and SBF transcription factor complexes [21,22]. The genes in the MBF/SBF regulon also include, among others, the proposed negative regulators of APC/C-Cdh1 activity: the G1 cyclins Cln1 and 2, S-phase cyclins Clb5 and 6, as well as Acm1, a stoichiometric inhibitor of APC/C-Cdh1 (see below) [23,24]. The architecture of the Start molecular network ensures minimizing cell-to-cell variability in timing of these events [25]; however, budding still occurs with substantial cell-to-cell variability [26].

Inactivation of APC/C-Cdh1 at cell cycle Start and reactivation during mitotic exit is mostly achieved through multisite phosphorylation and dephosphorylation of Cdh1 by CDK and a counteracting phosphatase Cdc14, respectively, at 11 CDK consensus sites [27]. Cdc14 antagonizes this CDK phosphorylation, promoting Cdh1 activation. Phosphorylated Cdh1 is unable to bind to the APC/C [27]. In addition, Msn5 mediates export of phosphorylated Cdh1 out of the nucleus, possibly promoting sequestration of the APC/C from nuclear substrates [28]. Cdh1 phosphorylation is essential for APC/C-Cdh1 inactivation and viability, even at Cdh1 expressed at endogenous levels [29]. B-type Clb3,4,5,6 cyclins have been suggested as cyclins promoting Cdh1 inactivation [10, 30], and genetic results [31] suggest possible additional involvement of *CLN* G1 cyclins.

The 11 consensus sites on *S*. *cerevisiae* Cdh1 are scattered throughout the protein. The 7 N-terminal sites are poorly conserved, suggesting a mechanism of inactivation by bulk negative charge, a common regulatory feature of CDK targets [32, 33]. In contrast, the 4 C-terminal sites are positioned in the WD40 domain and are well conserved. The WD40 domain, which has a critical role in substrate recognition [34], is not involved at the interface with APC/C [35, 36], making the four C-terminal sites unlikely to affect APC/C binding.

A recent high-resolution structure of the human APC/C-Cdh1 has provided insight into the mechanism of APC/C-Cdh1 regulation by Cdh1 phosphorylation [36]. The N-terminal unstructured domain of Cdh1 forms an interaction with the Apc1 and Apc8 subunits of the core APC [36] and phosphorylation at the N-terminal sites likely causes steric clashes and electrostatic repulsion [36]. These results support the bulk negative charge model of Cdh1 inactivation by phosphorylation.

Cdh1 is phosphorylated at many residues in addition to the 11 proposed CDK sites [37]. Phosphorylation of Cdh1 by both CDK as well as polo kinase Cdc5 was proposed to be required for mitotic spindle assembly [38], although mutation of Cdc5 binding sites and phosphorylation sites on Cdh1 had no obvious phenotype [29].

Recognition of substrates by APC/C-Cdh1 is blocked by a pseudosubstrate inhibitor Acm1 [23], providing a phosphorylation-independent control mechanism. Acm1 is expressed in a cell cycle-dependent manner at the Start transition. In addition, Acm1 is degraded at mitotic exit by APC/C-Cdh1 and possibly APC/C-Cdc20 [24]; however, other mechanisms might also regulate its degradation [39]. *ACM1* is not essential, suggesting that phosphorylation is sufficient for Cdh1 inactivation.

Recently, fluorescent reporters were used to study the dynamics and ordering of degradation of APC/C-Cdc20 targets during mitosis [40]. Here, we construct and validate a biosensor for APC/C-Cdh1 activity in the late G1 phase. We use it to measure the dynamics and cell-to-cell variability of APC/C-Cdh1 inactivation upon the Start transition, and to assess and quantify dependence of inactivation on Cdh1 phosphorylation sites, kinases and other regulators.

## Results

### A biosensor for APC/C-Cdh1 activity

**Fig 1:**
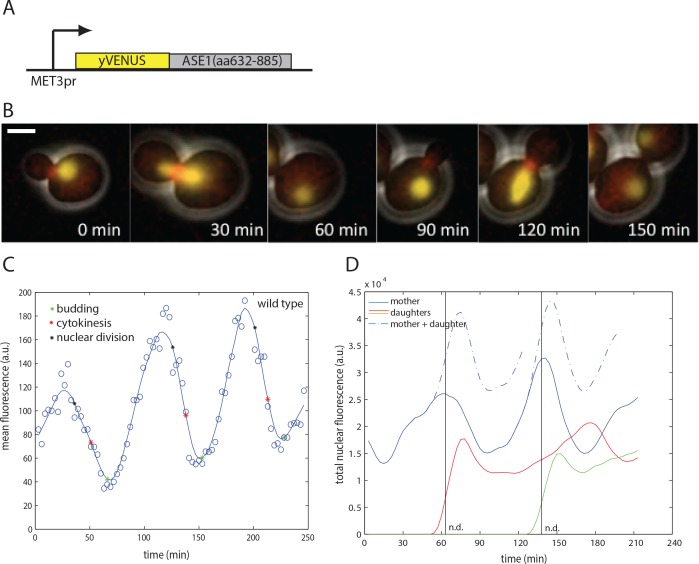
Biosensor for APC/C-Cdh1 activity. A) A scheme of the APC/C-Cdh1 biosensor construct. B) Images from a time lapse movie of exponentially growing wild type cells containing the biosensor (yellow) and a bud neck marker Myo1-mCherry (red) at indicated times. The biosensor was also used as a marker for nuclear division. Scale bar– 2 microns. C) Quantification of mean fluorescence in the cell body of a representative mother cell (smoothing spline fit, solid line; raw data, circles). D) Quantification of total nuclear fluorescence of two representative mother and daughter cell pairs (smoothing spline fit). n.d.—nuclear division.

In order to measure inactivation of APC/C-Cdh1 in single cells, we sought a fluorescent biosensor whose degradation would specifically require APC/C-Cdh1. Ase1, a spindle midzone protein, is strongly destabilized by APC/C-Cdh1, but not by APC/C-Cdc20 [13, 41]. A C-terminal Ase1 fragment (residues 632–885) was identified as the minimal fragment to support degradation by APC/C-Cdh1 [10]. We fused this fragment to the yellow fluorescent protein yVenus, under control of the *MET3* promoter (Fig 1A). This fragment imposes nuclear localization of the reporter, thus also providing a nuclear marker. The *MET3* promoter is expressed in absence of methionine at a constant level throughout the cell cycle [42]. yVenus was chosen as a fluorescent protein of choice due to its relatively fast maturation times.

We quantified cell fluorescence over the mother cell body in live-cell timelapse microscopy with semi-automated image segmentation software [42]. Fluorescence exhibits regular once-per-cell-cycle oscillations, rising around the time of bud emergence, and dropping around the time of nuclear division and cytokinesis (Fig 1B and 1C).

Since the Ase1-YFP fusion is nuclear-localized, an approximately two-fold drop is expected due to nuclear division (assuming equal segregation of nuclear components to mother and daughter), since our automated cell-tracking algorithm followed the mother cell body specifically. The drop was greater than twofold, and summing fluorescence over both mother and daughter did not eliminate the drop (Fig 1D); the excess decrease reflects cell-cycle-dependent degradation, which is rapid for about a 15-min interval around the time of cell division. This is approximately the time when Cdh1 is expected to be active, due to the drop in CDK activity after mitosis [27] (see Introduction).

**Fig 2:**
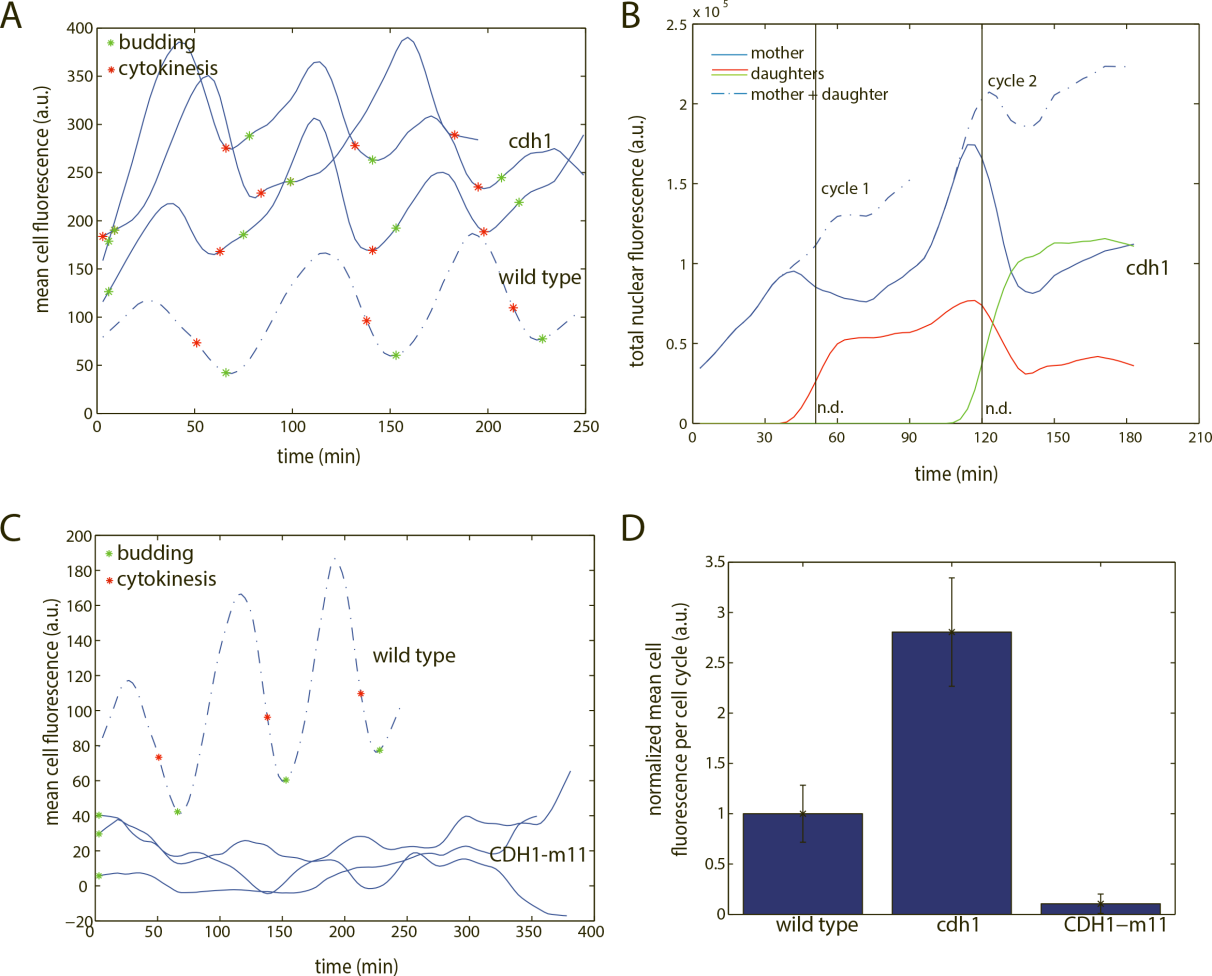
Characterization of the APC/C-Cdh1 biosensor. A) Quantification of mean cell fluorescence of three representative *cdh1* cells (smoothing spline fit). B) Quantification of total nuclear fluorescence of two representative mother and daughter *cdh1* cell pairs (smoothing spline fit). n.d.–nuclear division. C) Quantification of mean cell fluorescence of three representative *CDH1-m11* cells. D) Quantification of mean cell fluorescence, averaged across the cell cycle. Error bars represent standard error of the mean. n (wild type) = 9; n (*cdh1*) = 12; n (*CDH1-m11*) = 14.

To ask if Cdh1 was necessary for biosensor degradation, we measured biosensor fluorescence in *cdh1*-null cells. Mean biosensor fluorescence was on average three-fold elevated throughout the cell cycle in *cdh1* compared to wild type (Fig 2A and 2D). Fluorescence dropped rapidly just before cytokinesis (Fig 2A). This is likely due to nuclear division with loss of half the nuclear biosensor to the daughter, since the sum of total fluorescence intensity of mother and daughter cells reduced or eliminated this drop, unlike in wild type (Fig 2B). Thus biosensor degradation is dependent on Cdh1.

Inactivation of Cdc20 removes the other main activator of APC/C, and *cdc20*-blocked cells have high CDK activity, and consequently, inactive Cdh1 [27]. In *cdc20*-blocked cells, biosensor fluorescence was high and approximately constant for an extended period (S1 Fig).

To ask if constant APC/C-Cdh1 activity effectively eliminates the biosensor, we measured biosensor fluorescence in cells expressing *CDH1-m11*, an allele that lacks all 11 CDK phosphorylation sites, expressed from the endogenous *CDH1* locus [27,29]. Endogenous *CDH1-m11* expression causes a lethal block before mitotic entry, but cells can be conditionally kept alive by overexpressing *ACM1*, the stoichiometric inhibitor of APC/C-Cdh1, from a galactose-inducible promoter [29]. Upon switch to glucose to turn off *GAL-ACM1*, *CDH1-m11* cells arrested before mitotic entry, with low and constant biosensor fluorescence (Fig 2C and 2D), indicating continuous degradation. This result suggests that Cdh1, if unregulated by phosphorylation, is sufficient for elimination of the biosensor.

These results support Cdh1 specificity of the biosensor: Cdh1 is necessary for biosensor degradation, constant high APC/C-Cdh1 activity causes persistent degradation of the biosensor, and degradation is restricted to known times of Cdh1 activity, between mitosis and subsequent cell cycle initiation.

### Timing of APC/C-Cdh1 inactivation

We wanted to determine the timing of Cdh1 inactivation, compared to other cell cycle events. Due to slow maturation of the fluorescent protein, a method to assign a specific APC/C-Cdh1 turnoff time from gradually rising fluorescence traces was needed. We computed the first derivative of the smoothed fluorescence trace for the biosensor, and assigned APC/C-Cdh1 inactivation to be the time where the first derivative changed sign from negative to positive (i.e. where the slope turns upwards; S2A Fig). For biosensor data, this method was less sensitive to noise than the previously used second derivative-based method for promoter turn-on times [18].

We developed a computational method to assess the effects of experimental noise on the measurements of inactivation time (S2B–S2F Fig). The method is based on fitting the traces with a smoothing spline, then calculating measurement noise as deviations from the smoothing spline. We then created bootstrapped ‘replicates’ by adding random noise (with equal standard deviation as calculated) to the smoothing spline fit, and measuring apparent inactivation times from the simulated data (S2D Fig). By applying this procedure multiple times on each cell cycle, we found that the measurement in majority of cell cycles is very resistant to noise (S2E and S2F Fig), and allows identification and elimination of unreliable cycles from the pooled data. A residual tolerated error (3 minutes; the frame resolution of our movies) sets the upper limit to the contribution of measurement error, measured variability in excess of 3 min likely represents real biological variability.

(Note that we interpret the ‘noise-sensitive’ class of cell cycles, that are excluded from our data tabulations, as coming about for purely technical reasons, such as stochastic occurrence of significantly ‘wrong’ points at positions in the time course that are critical for the fitting. As best we can tell there is nothing overtly unusual about these cell cycles; we do not think we are excluding a specific biological class by this procedure).

As a reference time for Cdh1 inactivation we used nuclear export of the Whi5 transcriptional repressor. Whi5 is nuclear during approximately the same interval when Cdh1 is active (mitosis to Start). Whi5 nuclear exit is promoted by G1 cyclin-dependent positive feedback at Start [18]. Whi5 is kept out of the nucleus subsequently by S- and mitotic cyclin-CDK [43], and only re-enters the nucleus upon mitotic cyclin degradation during mitotic exit.

**Fig 3:**
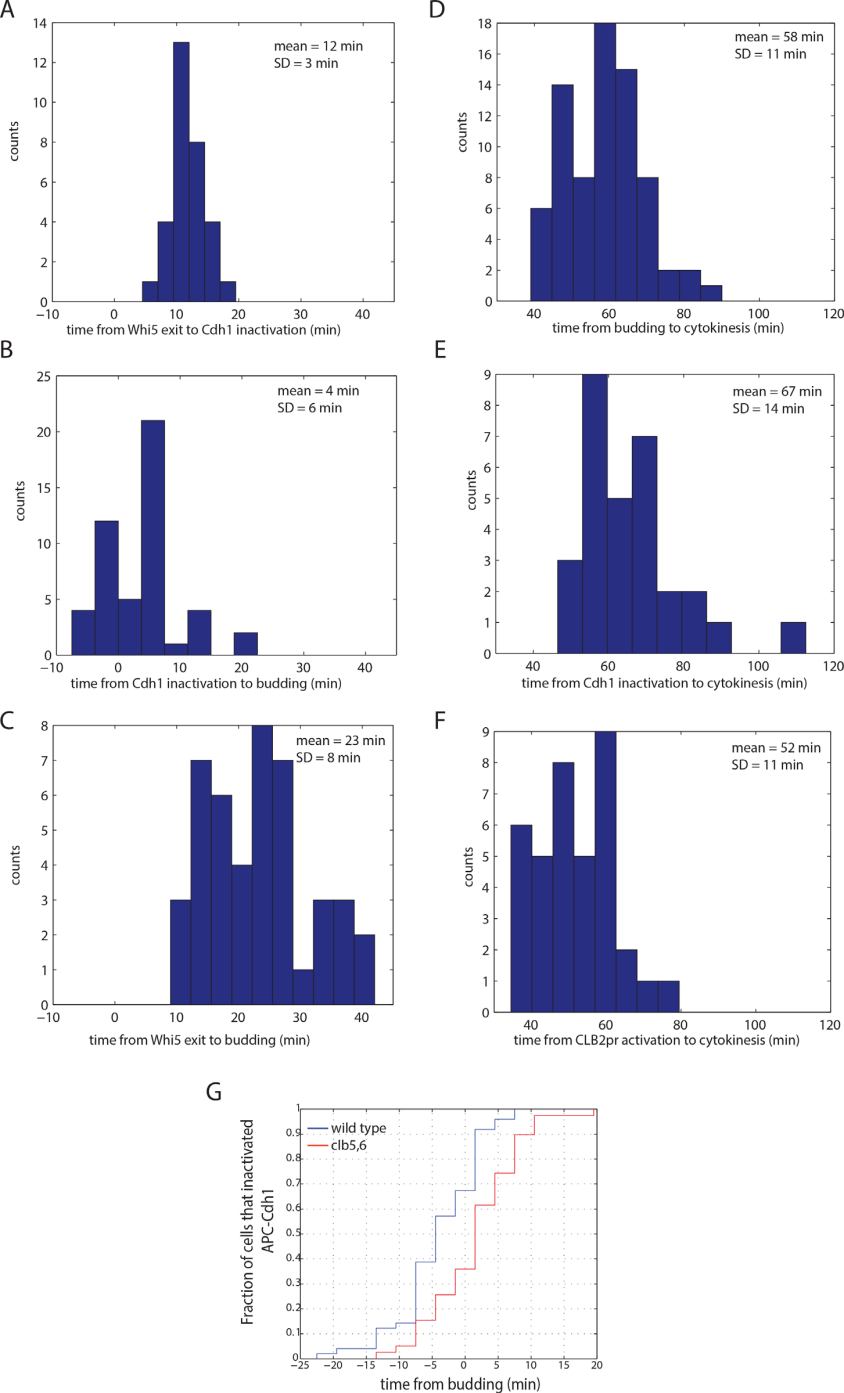
Timing of cell cycle intervals. A) A histogram of times from Whi5 nuclear exit to APC/C-Cdh1 inactivation. B) A histogram of times from budding to APC/C-Cdh1 inactivation. C) A histogram of times from Whi5 nuclear exit to budding. D) A histogram of times from APC/C-Cdh1 inactivation to subsequent cytokinesis. E) A histogram of times from budding to subsequent cytokinesis. F) A histogram of times from *CLB2* promoter turn-on to subsequent cytokinesis. In A-F, APC/C-Cdh1 inactivation and *CLB2* promoter turn on were determined from smoothing spline fits as shown in S2A Fig, and cell cycles in which the measurement was sensitive to noise were omitted. Both mother and daughter cells were pooled together. G) A cumulative distribution plot of APC/C-Cdh1 inactivation times with respect to bud emergence in wild type and *clb5*,*6* cells. n (wild type) = 55; n (*clb5*,*6*) = 47.

Using cells double-labeled with Whi5-GFP and the YFP biosensor, and quantitative deconvolution to eliminate crossover signal, we determined that APC/C-Cdh1 inactivation occurred 12+/-3 min after Whi5 nuclear exit (Fig 3A). The 3 min standard deviation of this measurement is equal to the upper estimate of the fitting error. Therefore, APC/C-Cdh1 inactivation occurs rapidly and with low cell-to-cell variability after Whi5 nuclear exit.

Separately, we measured the time of APC/C-Cdh1 inactivation with respect to budding, using Myo1-mCherry as a bud neck marker. On average, Cdh1 was inactivated 4 minutes before formation of the Myo1 ring at the bud neck (Fig 3B). However, there was considerable variability between cells, likely representing biological cell-to-cell variability in coherence between these two events. Variability of similar magnitude was noted between Whi5 exit and budding [26], (Fig 3C).

We also measured the time of APC/C-Cdh1 inactivation with respect to subsequent cytokinesis (detected by disappearance of the Myo1-mCherry signal at the bud neck; Fig 3E). This time was variable, and comparable in variability to that of the time from budding to cytokinesis (Fig 3D). These observations suggest highly variable birth-to-budding times, and independently highly variable budding-to-cytokinesis times, as reported previously [26], with the nearly invariant Whi5 exit-Cdh1 inactivation sequence temporally uncoupled from either.

S phase cyclins Clb5 and Clb6 were proposed to be major regulators of Cdh1 [10]. To assess their contribution to APC/C-Cdh1 inactivation as measured by the biosensor, we performed time lapse experiments and measured the time of inactivation of APC/C-Cdh1 in *clb5*,*6* cells with respect to budding. We find no morphological defects in *clb5*,*6* cells, and biosensor levels oscillated once per cell cycle with similar dynamics. However, the apparent inactivation of APC/C-Cdh1 is delayed on average by 6 minutes (Mann-Whitney p = 2.8*10^−4^) with respect to wild type cells (Fig 3G). This delay is consistent with previous measurements [10] and suggests that Clb5 and 6-CDK are required for timely inactivation of Cdh1, although other cyclin-CDKs must carry out sufficient phosphorylation of Cdh1 in absence of S-phase cyclins (since *clb5*,*6* cells are viable but *CDH1-m11* cells, with fully unphosphorylatable Cdh1, are not [29]. *CLB5* and *CLB6* promote timely entry into S phase [44,45], and S phase is delayed by ~30 min in *clb5*,*6* double mutants [45], a substantially greater delay than we observe in Cdh1 inactivation. This comparison suggests that other cyclins beside Clb5,6 can more easily inactivate Cdh1 than they can promote DNA replication, suggesting a higher degree of cyclin specificity for control of replication than for Cdh1 inactivation.

We were unable to quantitatively assess the effect of G1 cyclin deletion on APC/C-Cdh1 inactivation timing, because G1 cyclins also affect the timing of budding and Whi5 exit [18]–thus we lack a usable reference point.

In summary, we have shown that APC/C-Cdh1 is inactivated rapidly and with highly reliable timing after Start. In the following section, we assess how multiple regulatory mechanisms–multisite phosphorylation of Cdh1 by multiple cyclin-CDK complexes, inhibition by Acm1, and nuclear export by Msn5 –might provide such rapid and reliable inactivation.

### Multisite phosphorylation of Cdh1 is required for reliable APC/C-Cdh1 inactivation

**Fig 4:**
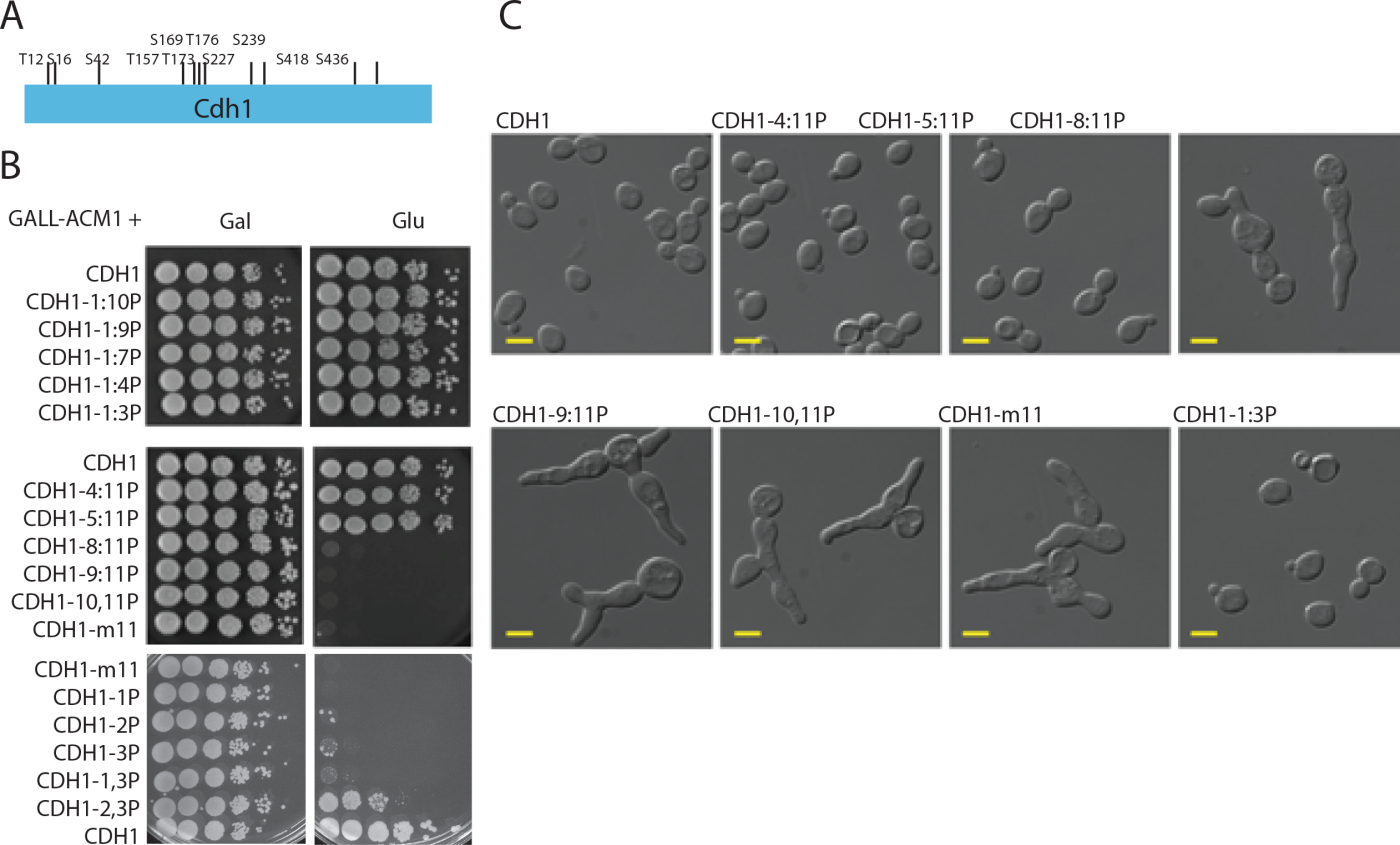
Multisite phosphorylation of Cdh1. A) Position of CDK consensus phosphorylation sites on Cdh1. B) tenfold serial dilutions of strains bearing indicated partially phosphorylatable *CDH1* alleles. The alleles are named by the sequential numbers of the phosphorylation site counting from the N-terminus that are retained (e.g. *CDH1-1*:*3P* contains the sites 1,2 and 3 but lacks the subsequent eight sites; *CDH1-1*,*3P* contains the sites 1 and 3 but lacks site 2 and sites 4–11.). C) DIC images of cells bearing the phosphorylation mutant alleles. Scale bar– 5 microns.

Cdh1 contains 11 putative CDK phosphorylation sites (Fig 4A). Previous work indicated that these sites might act in a generally additive manner [27], but exact contributions of specific sites were not determined. Additionally, these studies were performed by overexpression; notably, high-level overexpression of even wild-type *CDH1* is toxic [41]. In order to assess the importance of particular phosphorylation sites, we devised a strategy to construct series of partially phosphorylatable CDH1 alleles, starting with either the most N-terminal or C-terminal phosphorylation site, using a homologous recombination strategy (see Materials and Methods). These are exact gene replacements expressing Cdh1 at endogenous levels [29], thus avoiding issues of overexpression. We maintained partial phosphomutant alleles in the presence of *GAL-ACM1*, since overexpression of the inhibitor Acm1 rescues lethality of *CDH1-m11* [29]. To assess viability, cells were then plated onto glucose to turn off exogenous ACM1 expression (strains additionally contained endogenous *ACM1*).

Mutating up to 8 of the total 11 sites starting from the C-terminus had no effect on viability (Fig 4B). While mutation of up to 4 N-terminal sites was still compatible with viability, mutating 7 or more N-terminal sites resulted in complete block to colony formation on glucose medium (Fig 4B), and cells arrested with morphology similar to that seen with the completely unphosphorylatable *CDH1-m11* (Fig 4C), [29]. N-terminal sites may contribute more strongly to APC/C-Cdh1 inactivation, as fewer N-terminal sites are needed for viability. However, no specific phosphorylation site is essential, since viability can be achieved by non-overlapping subsets of the eleven sites (compare *CDH1-1*:*3P* to *CDH1-5*:*11P*).

We assessed the minimal requirements for phosphorylation sites among the three N-terminal sites. *CDH1* alleles containing only a single unmutated CDK phosphorylation site did not allow cell viability (Fig 4B). We also tested two of the three possible combinations with two sites from the three N-terminal sites. Among these, *CDH1-2*,*3P* allele (second and third sites present) resulted in partial viability: colony formation was only 1%-10% on glucose compared to galactose.

Cells with partially phosphorylatable *CDH1* alleles were morphologically defective, at varying frequencies. The phenotype seen in these cells is reminiscent of *CDH1-m11* cells, which uniformly arrest with elongated buds and periodically establish new buds (Fig 4C), [29]. Buds are elongated, probably due to inability to depolarize bud growth due to lack of mitotic Clb-CDK activity [46]. In contrast to *CDH1-m11*, where cells were uniformly abnormal with elongated and/or multiple buds, we observed ~20% of such abnormal cells in *CDH1-5*:*11P* cultures and 80–90% in *CDH1-2*,*3P* cultures.

### Reducing the number of phosphorylation sites on Cdh1 alters the kinetics of Cdh1 inactivation

**Fig 5:**
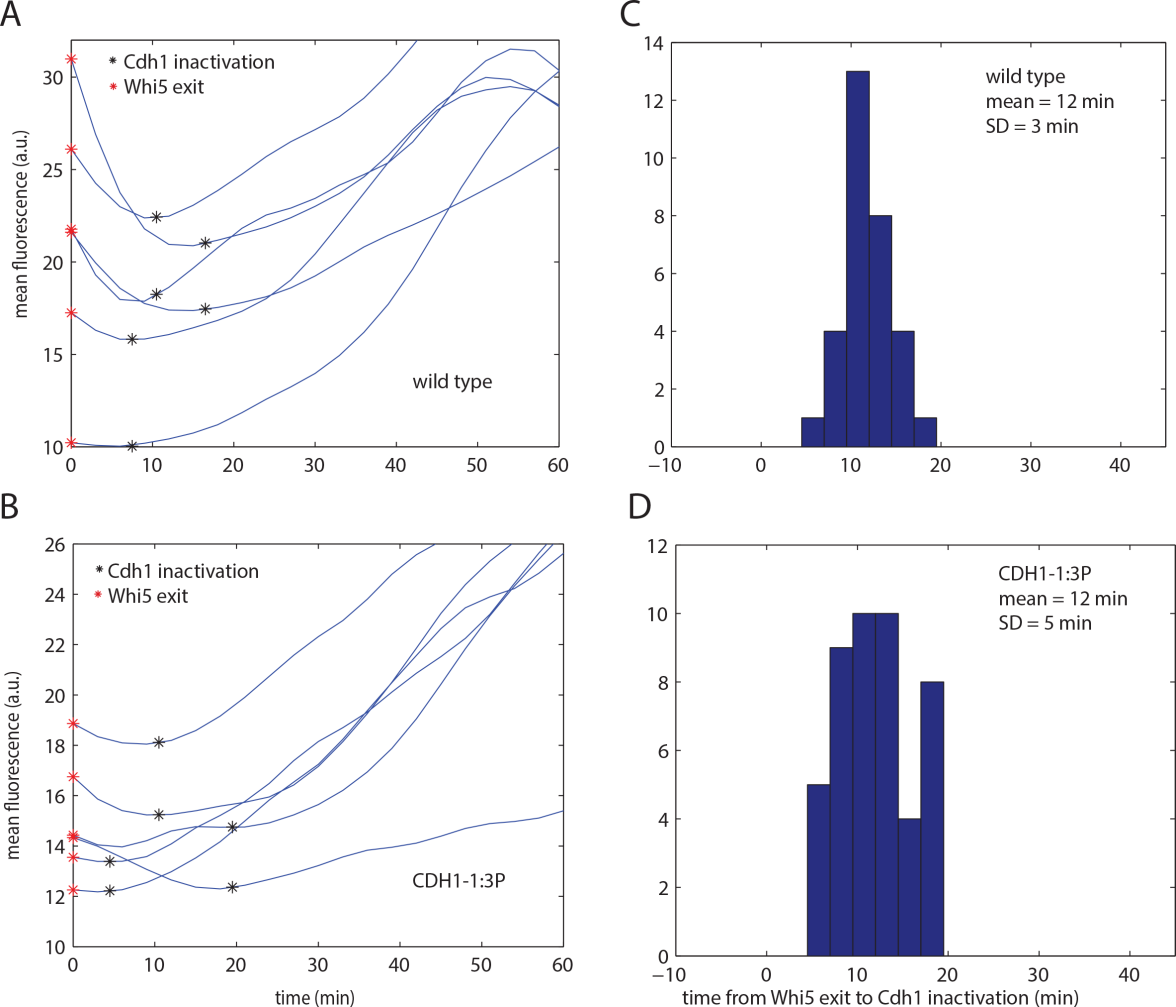
Cdh1 inactivation timing in *CDH1-1*:*3P* cells. A,B) Representative traces of biosensor fluorescence for wild type (A) and *CDH1-1*:*3P* (B). Each plot shows six representative traces, two each for 10 percentile, median and 90 percentile of the timing distribution population. C,D) A histogram of timing of Cdh1 inactivation with respect to Whi5 exit of the noise-filtered population for wild type (C; same data as Fig 3A) and *CDH1-1*:*3P* (D).

We hypothesized that multisite phosphorylation of Cdh1 could provide the reliability in timing of inactivation observed in Fig 3. To test this idea, we measured the time of Cdh1 inactivation with respect to Whi5 exit in the *CDH1-1*:*3P* mutant, identified above as containing the minimal number of sites required for complete viability and reliable cell cycle progression. We computed Cdh1 inactivation time with the same first derivative-based method as for wild type (Fig 5A and 5B), and applied the same method for filtering out noise-sensitive cell cycles (S2 Fig).

We estimated the degree of APC-Cdh1 inactivation in *CDH1-1*:*3P* and WT by the slope of the linear fit of raw data points between the minimum and maximum in the cell cycle (i.e. the interval when Cdh1 was inactivated) (S3C Fig). The average slope was lower in *CDH1-1*:*3P* than in wild type (two-tailed t test, P = 6.0*10^−5^) (S3D and S3E Fig). Although this method has limitations, including the assumption that Cdh1 remains inactivated to the same degree throughout the cycle, and the high degree of noise in the raw data, this result implies that APC/C-Cdh1 is incompletely inactivated in *CDH1-1*:*3P* cells compared to wild type.

We found that Cdh1 was on average inactivated with similar timing in *CDH1-1*:*3P* cells compared with wild type (12 minutes after Whi5 exit); however, the cell-to-cell variability was increased (standard deviation = 5 minutes, compared to 3 min for wild type) (Fig 5C and 5D). We note that the number of excluded cell cycles in *CDH1-1*:*3P* was higher than for wild type (45% compared to 27%), which could reflect the fact that inactivation time was more sensitive to noise due to more gradual inactivation.

To account for the fact that the filter based on noise sensitivity might skew the distribution by excluding too many data points, we also applied a different filter which was based on assuming a normal distribution of the population, and removed the most extreme outliers (S3A Fig). Lastly, we plotted all the results without excluding any cell cycles (S3B Fig). For wild type, the distributions of all three populations look similar; however, for *CDH1-1*:*3P*, filtering based on normal distribution is much less truncating than filtering based on noise sensitivity. However, regardless of the filtering method, variability of timing in *CDH1-1*:*3P* was higher that in wild type and statistically significant in all three datasets. We conclude that it is likely that the timing of inactivation of Cdh1 is more variable in *CDH1-1*:*3P* cells, although we cannot exclude the possibility that the observed apparent effect on variability is a statistical artifact due to more gradual Cdh1 inactivation in the mutant.

*CDH1-1*:*3P* exhibits the same mean timing of inactivation as WT, and the variability is greater in both directions. A potential explanation for this is that Cdh1-1:3P inactivation (complete or partial) is dependent on phosphorylation at a small number of sites; such a small number of phosphorylation events could by chance occur unusually early in some cells.

### Partially phosphorylatable Cdh1 leads to stochastic cell cycle arrest

Further reducing the number of phosphorylation sites lead to a partial defect in colony formation in *CDH1-2*,*3P*. To further characterize these partially penetrant phenotypes, we performed fluorescent time-lapse microscopy using strains with a bud neck marker, Myo1-mCherry, and the APC/C-Cdh1 biosensor, which also allowed for monitoring nuclear morphology and division.

**Fig 6:**
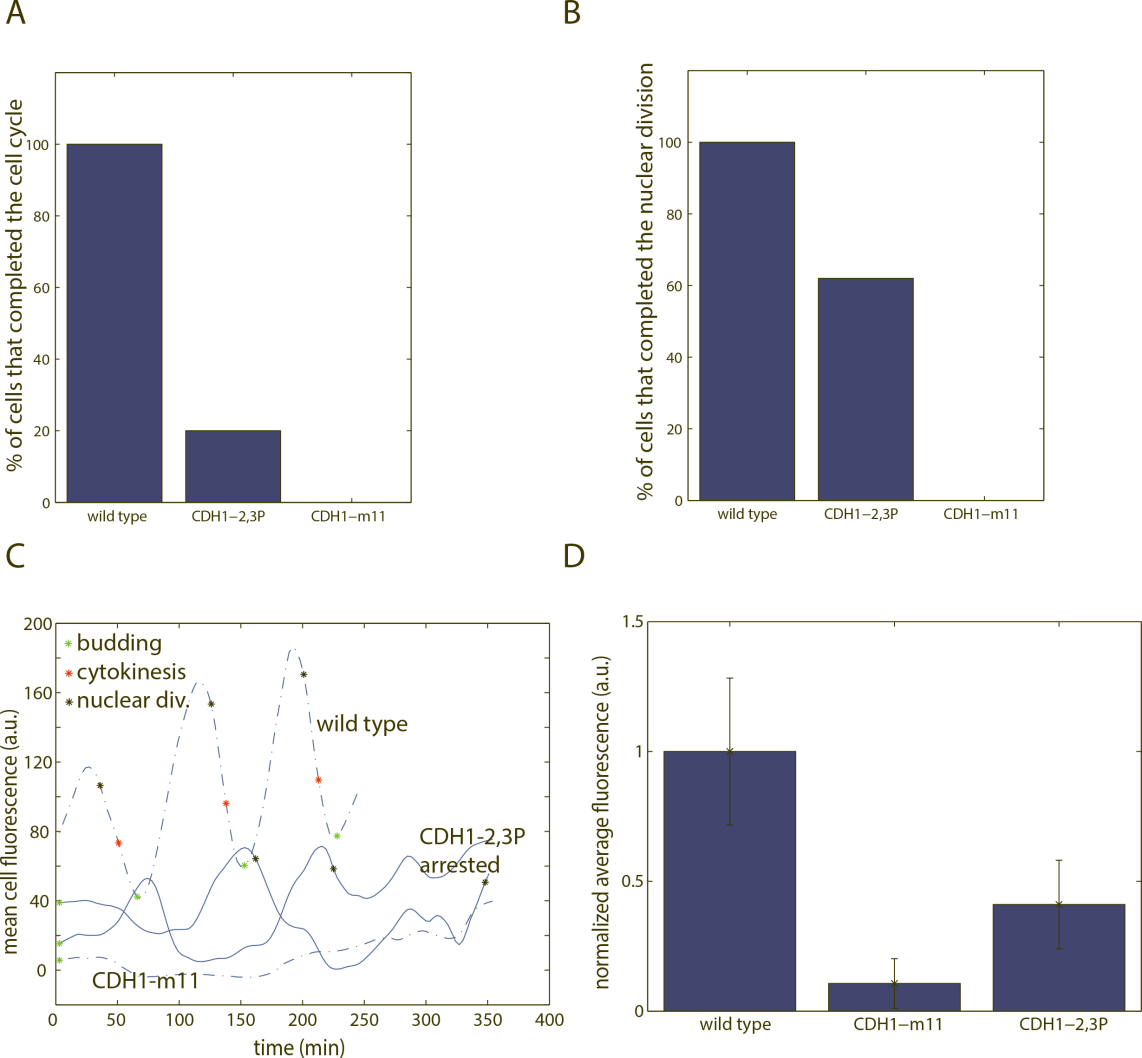
Time-lapse experiments of cells bearing *CDH1-2*,*3P* phosphomutant alleles. A) Fraction of cells that completed the first cell cycle. Completing the cell cycle was scored as completing cytokinesis before rebudding. The cells that did not score as completing the cell cycle typically grew elongated buds for an extended period of time and/or budded multiple times without performing cytokinesis. n (wild type) = 25; n (*CDH1-m11*) = 14; n (*CDH1-2*,*3P*) = 20. B) Fraction of cells that carried out at least one nuclear division during arrest. n (wild type) = 25; n (*CDH1-m11*) = 14; n (*CDH1-2*,*3P*) = 21. In A-B, cell cycle events were determined from time-lapse experiments, where cells were shifted from GAL-ACM1 on to off. We count the first cell cycle as the first cycle that a cell initiated as an unbudded cell in glucose to ensure complete removal of ectopic Acm1 (see materials and methods). C) Representative traces of biosensor fluorescence in *CDH1-2*,*3P* cells during arrest (smoothing spline fit). D) Quantification of mean cell fluorescence, averaged across the time period of arrest. Each data point represents average fluorescence throughout the period of arrest for one cell. Error bars represent standard error of the mean n (wild type) = 9; n (*CDH1-m11*) = 14; n (*CDH1-2*,*3P*) = 16.

*CDH1-m11 GAL-ACM1* cells uniformly arrested in the first cell cycle upon switch to glucose from galactose (Figs 4C, 6A and 6B) [29]. These cells grew elongated buds and frequently rebudded, but never underwent cytokinesis (Myo1-mCherry signal remained present at the bud site). In contrast, *CDH1-2*,*3P* cells were variable. 20% of the cells successfully completed the first cell cycle after switch to glucose (Fig 6A and 6B). Other cells formed elongated buds and rebudded multiple times, like *CDH1-m11* cells, without completing cytokinesis.

62% of arrested *CDH1-2*,*3P* cells ultimately performed at least one round of nuclear division (Fig 6B). However, in many cases these nuclear divisions were visibly aberrant–the nuclear mass separated unevenly. Nuclear divisions also occurred at very variable times throughout the arrest (shown on traces in Fig 6C).

In arrested *CDH1-2*,*3P* cells, the activity of APC/C-Cdh1 based on biosensor fluorescence was higher than in wild type but lower than in *CDH1-m11* (Fig 6C and 6D). This suggests that APC/C-Cdh1 activity is only partially restrained in *CDH1-2*,*3P* cells. Time-lapse microscopy using the mitotic cyclin Clb2, a known Cdh1 target, tagged with GFP, as well as immunoblotting against Clb2, gave similar results (S4 Fig).

*CDH1-2*,*3P* cells exhibited temporally variable biosensor levels. Drops in fluorescence only sometimes co-occurred with nuclear division (Fig 6C). This suggests that APC/C-Cdh1 activity in *CDH1-2*,*3P* cells undergoes oscillations in activity, perhaps due to cycles of phosphorylation and dephosphorylation of Cdh1-2,3P at the remaining two sites. While qualitatively apparent, oscillations were irregular; APC/C-Cdh1 inactivation times could not be scored reliably due to highly aberrant cell morphology, which strongly interfered with the cell segmentation software.

### Combinatorial regulation of Cdh1 by phosphorylation, Acm1 and Msn5

In addition to phosphorylation by CDK, multiple other redundant factors may contribute to Cdh1 inhibition. A stoichiometric inhibitor Acm1 is expressed in S and M phase and inhibits Cdh1-APC/C activity through pseudosubstrate inhibition [23,24]. In addition, Cdh1 is exported out of nucleus via the nuclear export factor Msn5 [28]; Msn5-Cdh1 interaction is phosphorylation-dependent. We sought genetic interactions with these factors, with the logic that if phosphorylation control is partially disrupted, these factors might become essential.

**Fig 7:**
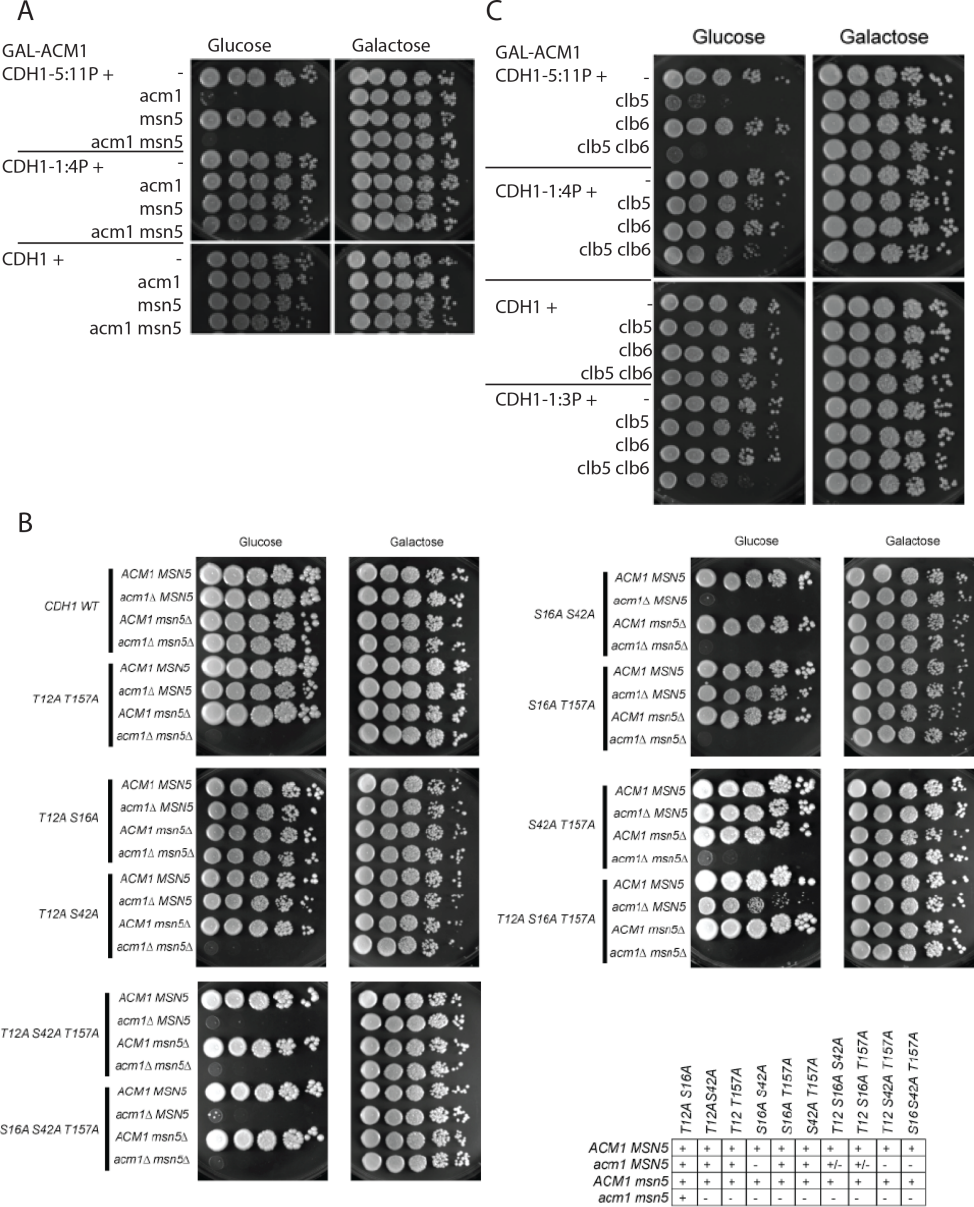
Tenfold serial dilutions of strains with indicated genotypes, all with *GAL-ACM1*, spotted on galactose and glucose plates. A) Genetic interactions between partial phosphomutants *CDH1-1*:*4P* and *CDH1-5*:*11P* and *acm1* and *msn5* deletion. B) Genetic interactions between selected phosphomutants with mutations among the four N-terminal sites (indicated in the left column) and *acm1* and *msn5* deletion. Table qualitatively summarizes the synthetic lethalities, with *CDH1* genotype on the x axis. ‘+’ is fully viable, ‘+/-’ is intermediate ability to form colonies, and ‘–’ is strictly inviable on glucose. C) Genetic interactions between partial phosphomutants *CDH1-1*:*3P*, *1*:*4P and 5*:*11P* and *clb5* and *6* deletions.

*ACM1* was required in the *CDH1-5*:*11P* background but not in the *CDH1-1*:*4P* background (Fig 7A). This result is consistent with the general greater potency of N-terminal phosphorylation sites for Cdh1 control in our assays. The more severe *CDH1-2*,*3P* mutation, with reduced viability even in the *ACM1* background, was completely inviable in combination with *acm1* deletion. In the presence of the 7 C-terminal sites, we tested nearly all combinations of N-terminal sites for viability in the *acm1* background (Fig 7B). Presence of site 2 alone, or of sites 1 and 3 together, or of sites 3 and 4 together, fully restored viability; presence of site 3 alone gave an intermediate result. As with functional mapping for viability in the *ACM1* background, these results suggest likely greater importance of the N-terminal sites, but no specific dependence on any single site, for viability in the *acm1* background. This is a plausible interpretation since Acm1 is not thought to depend on Cdh1 phosphorylation for binding [24].

*MSN5* was not required for viability in any *CDH1* phosphomutant background, provided *ACM1* was present. However, *acm1 msn5* double mutation resulted in inviability in cells expressing *CDH1* with mutations in various combinations of sites from among the N-terminal four (T12, S16, S42, T157, Fig 7B). In contrast, *msn5 acm1 CDH1-1*:*4P* (with only these four sites present) was fully viable (Fig 7A). This pattern of inviability in the *acm1 msn5* double mutant background suggests the speculation of preferential interaction of Msn5 with the 7 C-terminal Cdh1 phosphorylation sites (since effects of *msn5* deletion could only be detected in backgrounds with the C-terminal sites present). Msn5-Cdh1 interaction is thought to depend on Cdh1 phosphorylation [28], so some specificity in genetic interaction between *msn5* and phosphosite mutations might be expected.

Overall, these results suggest that Acm1 and Msn5 can act as safeguards for reliable APC/C-Cdh1 inactivation if phosphorylation of Cdh1 is incomplete in particular cell cycles.

### Genetic interaction of *CDH1* phosphosite mutations with *clb5*,*6* deletion

*CLB5* and *CLB6* are B-type cyclins expressed around the time of cell cycle Start [43,44], and Clb5,6 were proposed as important for inhibitory Cdh1 phosphorylation [10,30]. We found that *CLB5* was required for viability in the *CDH1-5*:*11P* background, but not in the *CDH1-1*:*4P* background (Fig 7C). This result could suggest specific interaction of Clb5 with the C-terminal sites. However, *CLB5* was also required in the *CDH1-2*,*3P* background, indicating that any such specificity was not absolute (S5 Fig). *CLB6* overlaps in timing of transcription and in functional assays with *CLB5* [45]. Single *clb6* deletion had no effect on viability of any *CDH1* phosphomutant background tested; however, when combined with *clb5* deletion, additional *clb6* deletion was semi-lethal in the *CDH1-1*:*4P* and *CDH1-1*:*3P* backgrounds. Overall, these results suggest that Clb5 and Clb6 may interact (directly or indirectly) with both N- and C-terminal sites of Cdh1 phosphorylation, consistent with the delay in APC/C-Cdh1 inactivation observed in *clb5*,*6* cells (Fig 3G).

### Genetic interaction of *CDH1* phosphosite mutations with *cln1*,*2* deletion

**Fig 8:**
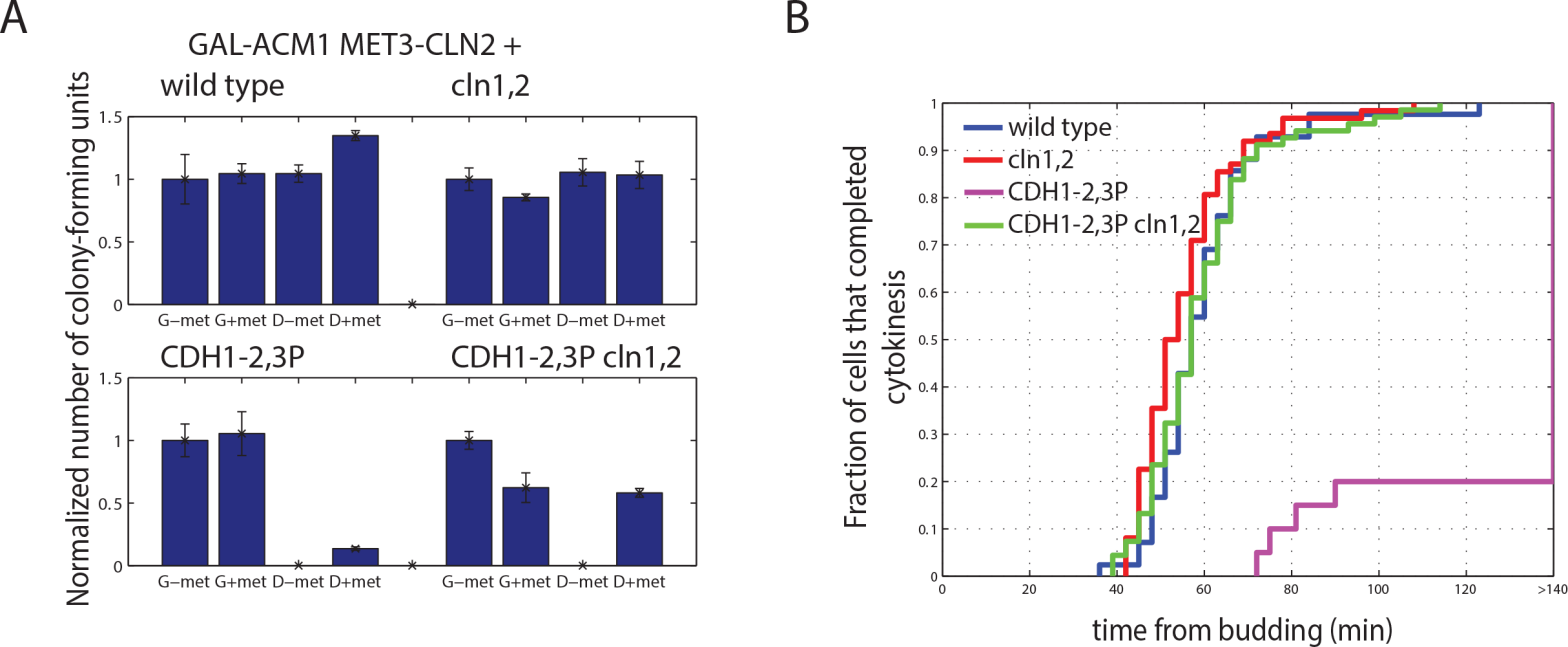
Genetic interactions of *CDH1* phosphomutants with G1 cyclin deletions. A) Quantification of number of colony forming units of strains bearing *CDH1-2*,*3P* and *cln1*,*2* deletions, normalized to colony number on G-met for each genotype. Error bars represent standard error of the mean from triplicates. B) Cumulative distribution of the budded period of the cell cycle of cells bearing *CDH1-2*,*3P* and *cln1*,*2* deletions, measured from time lapse experiments. Budding and cytokinesis were scored using the Myo1-mCherry bud neck marker. n (wild type) = 42; n (*cln1*,*2*) = 62; n (*CDH1-2*,*3P cln1*,*2*) = 68; n (*CDH1-2*,*3P*) = 20.

G1 cyclins *CLN1*,*2* are co-expressed with *CLB5*,*6* at Start, and genetic data suggests that *CLN1*,*2* contributes to Cdh1 inactivation [31]. We constructed strains with *cln1*,*2* deleted to see if removal of G1 cyclins might enhance phenotypes of *CDH1* phosphosite mutants, as observed above for *clb5*,*6*. *cln1*,*2* strains were made conditional with a *MET-CLN2* construct, so that effects of *cln1*,*2* deletion were restricted to the experimental conditions with methionine present to repress *MET-CLN2*. We found to our surprise that *cln1*,*2* deletion largely rescued the 10- to 100-fold defect in colony formation of *CDH1-2*,*3P* described above (Fig 8A, S6A Fig) and eliminated the abnormal elongated bud morphology (S6B Fig). Therefore, the absence of Cln1,2 may alleviate the requirement for Cdh1 phosphorylation. This alleviation was incomplete, as *cln1*,*2 CDH1-m11 GAL-ACM1* cells expressing completely unphosphorylatable Cdh1 were inviable on glucose).

Consistently, the duration of the budded period of the cell cycle was indistinguishable in *cln1*,*2 CDH1-2*,*3P*, *cln1*,*2*, and wild type (Fig 8B), compared to the strong block in *CDH1-2*,*3P*. Clb2 levels were not substantially restored in *CDH1-2*,*3P cln1*,*2* cells compared to *CDH1-2*,*3P* (S6C Fig); however, these are asynchronous averages, so an effect of *cln1*,*2* deletion on Clb2 accumulation restricted to specific cell cycle times cannot be ruled out.

Interestingly, we observed that constitutive expression of *CLN2*, by keeping *MET-CLN2* on, had the opposite effect to *cln1*,*2* deletion, strongly enhancing the lethal effect of *CDH1-2*,*3P* (Fig 8A, compare D+met and D-met).

These results were counterintuitive, since the simpler expectation was that deleting cyclins (reducing CDK kinase activity potentially directed against Cdh1) would interact negatively with reduction in Cdh1 phosphosite number (as with *clb5* deletion described above). *cln1*,*2* deletion also did not interact negatively (and may interact positively) with a different *CDH1* phosphosite mutant, *CDH1-5*:*11P*, (S6D Fig) which, in contrast, was lethal in the absence of *clb5* (see above). Thus, according to these genetic assays, Clb5, but not Cln1 and Cln2, exhibits behavior expected for a major Cdh1-regulatory kinase.

In the *cln1*,*2* background, SBF- and MBF-targeted genes are delayed and asynchronous in transcriptional onset, but once activated they are expressed for a prolonged time compared to wild type [18]. We speculate that this may explain rescue of *CDH1* phosphomutants by *cln1*,*2* deletion: *ACM1* and *CLB5* are both in the SBF/MBF gene set, and their prolonged or elevated expression might help inactivate Cdh1. Consistent with this explanation, *cln1*,*2* deletion did not rescue inviability of *CDH1-5*:*11P acm1* (data not shown).

## Discussion

We developed an assay to study the dynamics of APC/C-Cdh1 activity during the cell cycle. Inactivation of APC/C-Cdh1 after Start occurs with very reliable timing. Multiple kinases, phosphorylation sites, Acm1 and Msn5 contribute redundantly to this inactivation. Cdh1 inactivation is due to a highly redundant system with no uniquely required element.

Early work on Sic1 led to the proposal of a simple counting mechanism for multisite phosphorylation [47], although subsequent work found out different roles for different phosphorylation sites [48]. A counting mechanism was also proposed for Ste5, where protein phosphorylation was shown to disrupt interactions with the plasma membrane by introducing negative charge [32]. Our results suggest some differentiation among the 11 CDK sites on Cdh1, ruling out a simple counting mechanism. For example, *CDH1-1*:*3P* with only three sites remaining results in full viability, while *CDH1-8*:*11P* with four sites results in complete inviability. The strength of a particular phosphorylation site on Cdh1 likely depends on the location of the residue in the structure. It is not possible at present to cleanly line up individual sites with specific genetic requirements (e.g. for *ACM1* or *CLB5*).

Whi5 nuclear exit marks (and in large part controls) onset of Start-specific transcription. This simple causal relationship may in part explain rapid Cdh1 inactivation upon Whi5 nuclear exit, since the Start regulon includes the important Cdh1 regulators *CLB5*,*6* and *ACM1*. A subsequent important cell cycle event, activation of the promoter for the mitotic cyclin *CLB2*, occurs within about 10 min of Cdh1 inactivation (Fig 3F). This also may be a causal relationship: transcription of *CLB2* and other members of the *CLB2*-cluster of mitotic genes is controlled by Clb2-dependent positive feedback [49], so stabilization of Clb2 by Cdh1 inactivation is likely required to initiate the loop. Additionally, in a possible independent requirement for Cdh1 inactivation for this loop, Cdh1 promotes degradation of the *CLB2*-cluster transcription factor Ndd1 [16]. Consistently, *CDH1-m11* cells are defective in activation of the *CLB2* promoter (data not shown).

It is interesting that this accounting leaves unexplained the substantial time variability of the budded period of the cell cycle [26]; from our results, this variability must follow Cdh1 inactivation, and likely also follows activation of the *CLB2* cluster of mitotic transcription.

Inactivation of APC/C-Cdh1 is an essential step for the nuclear division cycle [29], since it is required for expression and stabilization of mitotic cyclins and spindle proteins. Therefore, sporadic delays in APC/C-Cdh1 inactivation could be rate-limiting for subsequent cell cycle events. The architecture of the Start molecular network may have evolved in part to suppress noise in inactivation of APC/C-Cdh1. Budding, which is more variable in timing than Cdh1 inactivation, is essential but may have a broader time window in which it can occur without negative consequences. This is likely achieved through the morphogenesis checkpoint [50], which ensures maintaining the proper ordering of cell cycle events.

APC/C-Cdh1 regulators are redundant on multiple levels: phosphorylation sites on Cdh1, kinases that can phosphorylate these sites, and inhibition by Acm1 and Msn5. Significant disruption of the APC/C-Cdh1 inactivation mechanisms results in either a delay in APC/C-Cdh1 inactivation, or stochastically aberrant cell cycle progression. Incomplete phosphorylation of Cdh1 greatly increased cell-to-cell variability in timing and extent of Cdh1 inactivation, in contrast with the rapid and reliable inactivation of APC/C-Cdh1 in wild type cells.

## Materials and methods

### Strains and plasmids

Standard methods were used throughout. All strains are W303-congenic. The Cdh1 biosensor was constructed by amplifying the *ASE1* degron sequence from the plasmid PB1452 (a gift from David Pellman) and cloned into pGC25 plasmid (a gift of Gilles Charvin) to obtain the biosensor plasmid, which was then integrated into the *TRP1* locus. The *CDH1* phosphomutants *CDH1-1C – 8C* and *3N-11N* strains were constructed by a modification of the method described in [29]. Briefly, a strain was constructed with a tandem duplication of *cdh1* and *cdh1-m11*, interrupted by selectable markers *LEU2* and *TRP1*, respectively (inserted at different locations within *CDH1* coding sequence), with an *URA3* marker in between. Upon 5-FOA selection, we screened for leu- trp- recombinants, which yielded a series of mutants depending on the exact region of homologous recombination. The recombinants were identified by sequencing the PCR product. The single and double phosphomutants were constructed by integrating an *URA3* plasmid bearing the desired *CDH1* mutant into the *CDH1* locus and subsequent 5-FOA selection. The mutation was confirmed by sequencing the PCR product. The phosphomutants were generated in either *GAL-ACM1* background or in *cdc23-1* background and subsequently crossed into *GAL-ACM1 CDC23* background.

### Culture conditions

Standard reagents and media were used throughout. Cells were grown either in synthetic medium without methionine and 2% glucose or 3% galactose (D/G-met) or rich YEP medium with 2% glucose or 3% galactose (D/G). DIC imaging, immunoblotting and bud counts of *CDH1* phosphomutants were performed at the 6 hour time point after exposure to glucose.

### Fluorescence time-lapse microscopy

Time-lapse imaging was performed using the instrumentation and software as described previously [42], except that cells were grown in a microfluidic chamber (CELLAsic Inc.) as per manufacturer instructions. Cultures were grown until early exponential phase before loading into the microfluidic chamber. Synthetic medium was used for all time-lapse experiments to minimize autofluorescence. Images were acquired every 3 minutes. In the experiments with *CDH1* phosphomutants, cells were pre-grown in the chamber into microcolonies in galactose (*GAL-ACM1* on) and then switched to glucose (*GAL-ACM1* off. Acm1 is unstable during G1 and stabilized in phosphorylated form [24], and depletion of ectopic Acm1 is very rapid in G1. Therefore, cells already budded at the time of switch to galactose are protected for the rest of the cell cycle, but enter the subsequent cell cycle without residual exogenous Acm1. The first cell cycle initiated as an unbudded cell in glucose was counted as the first cycle.

### Image analysis

Image processing and analysis was performed with Matlab. Image segmentation was done as described previously [42]. Budding and cytokinesis times were scored manually by appearance and disappearance of Myo1-mCherry foci. Nuclear divisions were scored manually in the yellow channel, making use of the fact that the APC/C-Cdh1 biosensor localizes in the nucleus. Fluorescence time series were smoothed by loess fitting over the 30 minutes timescale. Fluorescence values of unlabeled cells were subtracted. For the experiments where both the APC/C-Cdh1 biosensor and Whi5-GFP were used, fluorescence values were recalculated to correct for cross-bleeding between the fluorescence channels. Nuclei were detected either automatically using software described in [19] or manually.

### DIC microscopy

DIC images were taken using an Axioplan 2 microscope (Carl Zeiss, Thornwood, NY) and a 63x NA 1.4 Plan APO objective. The camera and the microscope were controlled by OpenLab software.

### Immunoblotting

Immunoblots were performed using standard protocols. The antibodies (rabbit polyclonal anti-Clb2 and anti-Pgk1 (Invitrogen, Carlsbad, CA)), were used in 1:10000 concentrations. Enhanced chemiluminescence signal was measured with DarkBox (Fujifilm, Greenwood, SC) with a charge-coupled device camera and quantified using MultiGauge software (Fujifilm). Quantification was performed using ImageJ. Intensities were corrected for background and normalized to the Pgk1 signal intensity.

## Supporting Information

S1 Fig Quantification of mean biosensor fluorescence in *cdc20* cells (smoothing spline fit). (EPS)

S2 Fig A) A representative trace of mean biosensor fluorescence along with computed first derivative. B) Difference between the smoothing spline fit and raw data. C) Histogram of difference between the smoothing spline fit and raw data at individual time points. D) A method for analyzing the sensitivity to measurement noise on a representative fluorescence trace. E,F) Examples of measurement sensitivity analysis for two cell cycles: smoothing spline fit and raw data, and histograms of measurement error (inset). E) represents a robust measurement, F) is a noise sensitive measurement.
(EPS)

S3 Fig A) A histogram of timing of Cdh1 inactivation for the normal-filterted population of wild type (top) and *CDH1-1*:*3P* (bottom). B) A histogram of timing of Cdh1 inactivation for the unfiltered population of wild type (top) and *CDH1-1*:*3P* (bottom). C) Illustration of the method for determination of fluorescence accumulation slope for the biosensor fluorescence on a sample wild type trace. D,E) A histogram of fluorescence accumulation slopes for wild type (D) and *CDH1-1*:*3P* (E).
(EPS)

S4 Fig A) Representative traces of Clb2-GFP fluorescence in *CDH1-2*,*3P* cells, along with wild type and *CDH1-m11*. B) Western blotting for Clb2 levels. Pgk1 was used as a loading control. Cultures were grown in glucose for 6 hours before samples were taken.
(EPS)

S5 Fig Tenfold serial dilutions of strains with indicated genotypes, all with *GAL-ACM1*, spotted on galactose and glucose plates. Partial viability of *CDH1-2*,*3P* is dependent on *CLB5* and *ACM1*, but not *MSN5* or *CLB6*.
(EPS)

S6 Fig A) Tenfold serial dilution of strains bearing *CDH1-2*,*3P* and *cln1* and *2* deletions. B) Microscopic images of strains bearing *CDH1-2*,*3P* and *cln1*,*2* deletions in both D-met (MET3-CLN2 on) and D+met (MET3-CLN2 off). Scale bar– 5 microns. C) Western blotting for Clb2 levels in strains from A). Cultures were grown in D+met media for 6 hours before sample preparation. Error bars represent standard error from 3 biological replicates. D) Fraction of unbudded (u), budded (b) and long-budded (lb) cells in cultures of strains bearing *CDH1-5*:*11P* and *cln1*,*2* deletions.
(TIFF)

S1 Table List of strains used in this work. (DOCX)
