# Supplementary figures and images for: An APC/C-Cdh1 Biosensor Reveals the Dynamics of Cdh1 Inactivation at the G1/S Transition
Source: PLoS One. 2016 Jul 13;11(7):e0159166. doi: 10.1371/journal.pone.0159166 (PMC4943722; doi:10.1371/journal.pone.0159166)

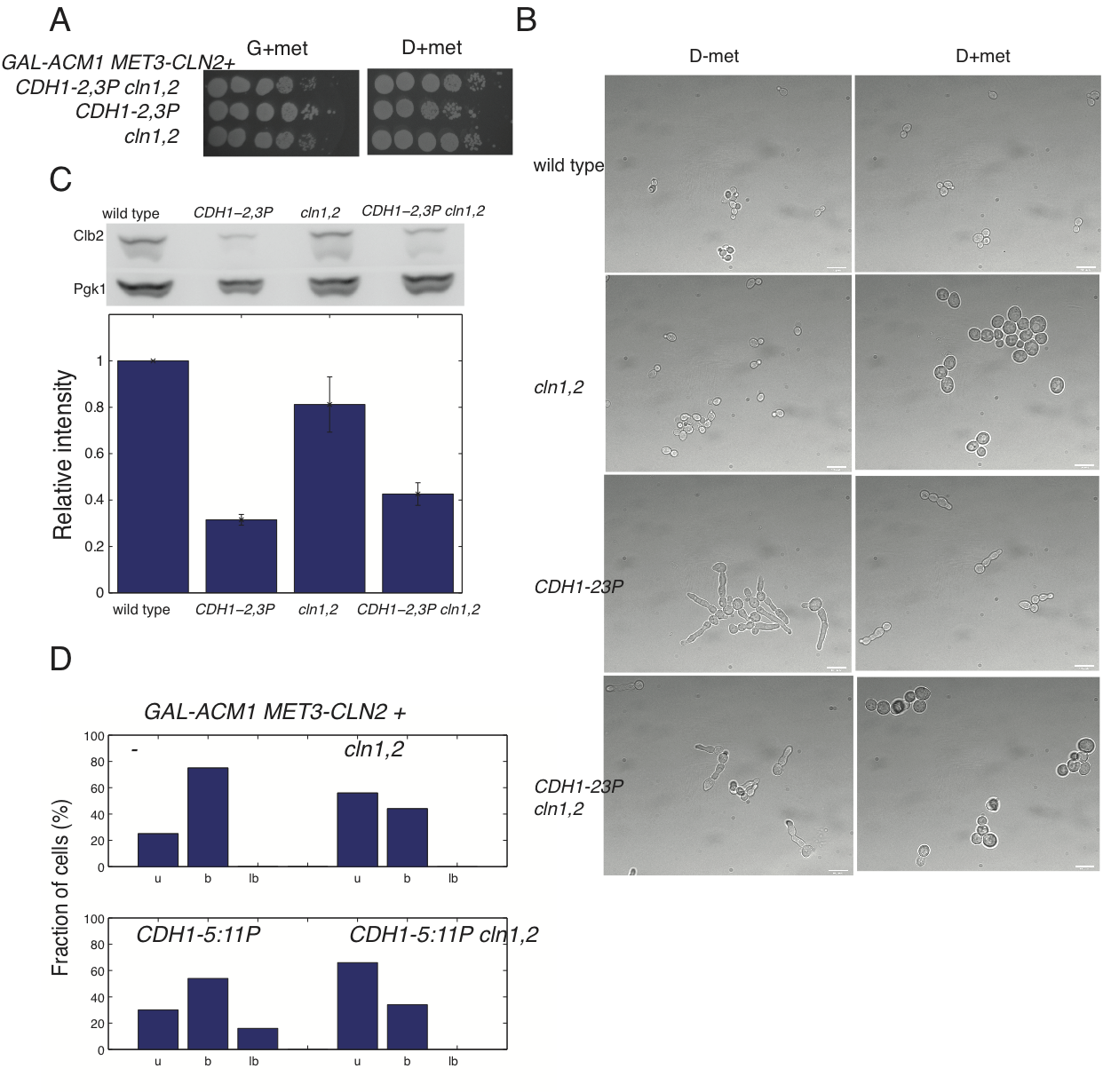

Supplement: S6 Fig — A) Tenfold serial dilution of strains bearing CDH1-2,3P and cln1 and 2 deletions. B) Microscopic images of strains bearing CDH1-2,3P and cln1,2 deletions in both D-met (MET3-CLN2 on) and D+met (MET3-CLN2 off). Scale bar– 5 microns. C) Western blotting for Clb2 levels in strains from A). Cultures were grown in D+met media for 6 hours before sample preparation. Error bars represent standard error from 3 biological replicates. D) Fraction of unbudded (u), budded (b) and long-budded (lb) cells in cultures of strains bearing CDH1-5:11P and cln1,2 deletions. (TIFF) [file pone.0159166.s006.tiff]
