# Supplementary material for: An APC/C-Cdh1 Biosensor Reveals the Dynamics of Cdh1 Inactivation at the G1/S Transition
Source: PLoS One. 2016 Jul 13;11(7):e0159166. doi: 10.1371/journal.pone.0159166 (PMC4943722; doi:10.1371/journal.pone.0159166)
Supplement: S1 Table — (DOCX) [file pone.0159166.s007.docx]

**S1 Table: List of strains used in this work.**

| GC46-03 | MATa MYO1-mCherry-HIS5 |
| --- | --- |
| AO832-3c | MATalpha MET3pr-yVENUS-ASE1deg-TRP1::trp1 MYO1-mCherry-HIS5 |
| AO852-2d | MATalpha MET3pr-yVENUS-ASE1deg-TRP1::trp1 MYO1-mCherry-HIS5 cdh1::LEU2 |
| AO95-4a | MATalpha MET3pr-yVENUS-ASE1deg-TRP1::trp1 MYO1-mCherry-HIS5 GALL-ACM1-LEU2::leu2 CDH1-m11 |
| AO261-5b | MATa MET3pr-yVENUS-ASE1deg-TRP1::trp1 WHI5-GFP-KanMX HTB2-mCherry-HIS5 |
| AO1221-12c | MAT? MET3pr-yVENUS-ASE1deg-TRP1::trp1 MYO1-mCherry clb5::HIS3 clb6::KanMX |
| JRC180d-6d | MAT? GAL1-ACM1-URA3::ura3 CDH1-1:10P |
| JRC182D-7b | MAT? GAL1-ACM1-URA3::ura3 CDH1-1:9P |
| JRC179C-4b | MAT? GAL1-ACM1-URA3::ura3 CDH1-1:7P |
| JRC181B-7c | MATalpha GAL1-ACM1-URA3::ura3 CDH1-1:4P |
| JRC135-10b | MATa GAL1-ACM1-URA3::ura3 CDH1-1:3P |
| JRC397A-4a | MATa GAL1-ACM1-URA3::ura3 |
| JRC177A-3d | MAT? GAL1-ACM1-URA3::ura3 CDH1-4:11P |
| JRC113-4b | MATa GAL1-ACM1-URA3::ura3 CDH1-5:11P |
| MNX32-6a | MATa GAL1-ACM1-URA3::ura3 CDH1-8:11P |
| JRC115-3a | MATa GAL1-ACM1-URA3::ura3 CDH1-10,11P |
| JRC116-6a | MATa GAL1-ACM1-URA3::ura3 CDH1-11P |
| JRC117-1b | MATa GAL1-ACM1-URA3::ura3 CDH1-m11 |
| AO7-1p1 | MATa GALL-ACM1-LEU2::leu2 CDH1-1P |
| AO7-2p2 | MATa GALL-ACM1-LEU2::leu2 CDH1-2P |
| AO7-3p3 | MATa GALL-ACM1-LEU2::leu2 CDH1-3P |
| AO17-13P1 | MATa GALL-ACM1-LEU2::leu2 CDH1-1,3P |
| AO17-23P | MATa GALL-ACM1-LEU2::leu2 CDH1-2,3P |
| 3149-21 | MATa GALL-ACM1-LEU2::leu2 CDH1-m11 |
| AO761-8b | MATa GALL-ACM1-LEU2::leu2 CDH1-2,3P MYO1-mCherry-HIS5 MET3-yVENUS-ASE1deg-TRP1::trp1 |
| AO812-12c | MATa GALL-ACM1-LEU2::leu2 CDH1-2,3P MYO1-mCherry-HIS5 CLB2-GFP-HIS5 |
| AO87-4c | MATa GALL-ACM1-LEU2::leu2 CDH1-m11 MYO1-mCherry-HIS5 CLB2-GFP-HIS5 |
| AO86-6b | MATa MYO1-mCherry-HIS5 CLB2-GFP-HIS5 |
| JRC397A-1c | MATa GAL1-ACM1-URA3::ura3 acm1::KanMX msn5::HIS3 |
| JRC397A-9b | MATa GAL1-ACM1-URA3::ura3 acm1::KanMX |
| JRC397A-9b | MATa GAL1-ACM1-URA3::ura3 msn5::HIS3 |
| MNX33-1d | MATa GAL1-ACM1-URA3::ura3 CDH1-5:11P acm1::KanMX msn5::HIS3 |
| MNX33-3c | MATa GAL1-ACM1-URA3::ura3 CDH1-5:11P msn5::HIS3 |
| MNX33-7d | MATa GAL1-ACM1-URA3::ura3 CDH1-5:11P acm1::KanMX |
| AO88-10a | MATa GALL-ACM1-LEU2::leu2 CDH1-2,3P acm1::KanMX msn5::HIS3 |
| AO88-7c | MATa GALL-ACM1-LEU2::leu2 CDH1-2,3P acm1::KanMX |
| AO88-10b | MATalpha GALL-ACM1-LEU2::leu2 CDH1-2,3P msn5::HIS3 |
| AO89-7b | MATa GALL-ACM1-LEU2::leu2 CDH1-2,3P clb6::KanMX clb5::HIS3 |
| AO89-10a | MATa GALL-ACM1-LEU2::leu2 CDH1-2,3P clb6::KanMX |
| AO89-11b | MATalpha GALL-ACM1-LEU2::leu2 CDH1-2,3P clb5::HIS3 |
| JRC437A-6a | MATa GAL1-ACM1-URA3::ura3 CDH1-5:11P clb6::KanMX clb5::HIS3 |
| JRC437A-4d | MATa GAL1-ACM1-URA3::ura3 CDH1-5:11P clb5::HIS3 |
| JRC437A-9c | MATa GAL1-ACM1-URA3::ura3 CDH1-5:11P clb6::KanMX |
| JRC436A-6c | MATa GAL1-ACM1-URA3::ura3 clb5::HIS3 |
| JRC436A-1c | MATa GAL1-ACM1-URA3::ura3 clb6::KanMX |
| JRC436A-1d | MATa GAL1-ACM1-URA3::ura3 clb6::KanMX clb5::HIS3 |
| AO1192-9a | MATa GALL-ACM1-LEU2::leu2 MET3-CLN2-TRP1::trp1 MYO1-mCherry-HIS5 |
| AO1194-6b | MAT? GALL-ACM1-LEU2::leu2 MET3-CLN2-TRP1::trp1 cln1 cln2 MYO1-mCherry-HIS5 |
| AO939-7b | MATa GALL-ACM1-LEU2::leu2 MET3-CLN2-TRP1::trp1 CDH1-2,3P |
| AO939-7a | MATa GALL-ACM1-LEU2::leu2 MET3-CLN2-TRP1::trp1 CDH1-2,3P cln1 cln2 MYO1-mCherry |
| AO1017-3d | MAT? GAL1-ACM1-URA3::ura3 MET3-CLN2-TRP1::trp1 CDH1-5:11P cln1 cln2 |
| AO1012-10b | MAT? GAL1-ACM1-URA3::ura3 MET3-CLN2-TRP1::trp1 CDH1-5:11P |
| AO365-2b | MATalpha CDH1-8C MET3-venus-Ase1deg-TRP1 HTB2-mCh-s.p.HIS5 WHI5-GFP-KanMX |
